# Impact of healthcare restrictions due to COVID-19 on early pregnancy complications: A cross-sectional study

**DOI:** 10.5339/qmj.2025.43

**Published:** 2025-06-30

**Authors:** Idriss Gharbi, Yasser Abdelaal, Moayyad Younis, Fathima Minisha, Obe John Ame, Victor Olagundoye, Thomas Farrell

**Affiliations:** 1 Department of Obstetrics and Gynecology, Women’s Wellness and Research Center, Hamad Medical Corporation, Doha, Qatar; 2 Department of Research, Women’s Wellness and Research Center, Hamad Medical Corporation, Doha, Qatar; 3 College of Medicine, Qatar University, Doha, Qatar *Email: volagundoye@hamad.qa

**Keywords:** COVID-19 pandemic, early pregnancy, ectopic pregnancy, miscarriage, complications, length-of-hospital stay

## Abstract

**Background:**

In March 2020, the World Health Organization declared COVID-19 a global pandemic. Healthcare organizations across the world introduced various measures to restrict the spread of the disease, with an increasing reliance on telephonic consultations as a key measure to limit exposure to COVID-19 in hospital facilities. This study assesses the impact of restrictive measures on gynecological emergency services by comparing services before the COVID-19 pandemic with services during the first and second waves of the pandemic (COVID-19 Peak 1 and COVID-19 Peak 2).

**Method:**

This was a retrospective single-center cross-sectional study comparing the first 50 women attending the emergency department (ED) of the Women’s Wellness and Research Center in Qatar with a gynecological complaint during three distinct periods. The peak of the first COVID-19 wave from June 2020 was considered COVID-19 Peak 1, and the peak of the second wave from April 2021 was COVID-19 Peak 2. The control group included 50 women who attended the ED during non-COVID-19 times. Early pregnancy complications (miscarriage and ectopic pregnancy) were compared between the three periods to determine the impact of the COVID-19 restrictions on the clinical presentation, subsequent management, and any patient safety issues arising out of this in terms of complications.

**Results:**

Data from 50 patients were analyzed during each study period (total = 150). There were no statistically significant differences in age, nationality, and parity between the three groups. The gestational age at diagnosis of ectopic pregnancy or miscarriage was significantly higher, 12.4 ± 4.0 weeks during COVID-19 Peak 1 compared to 10.9 ± 3.6 in pre-COVID-19 and 9.7 ± 3.9 in COVID-19 Peak 2 (*p* = 0.002). The length of hospital stays (median ± interquartile range) for women with the diagnosis of miscarriage was significantly shorter during COVID-19 Peak 1(1 ± 2 days) compared to pre-COVID-19 (2 ± 1.5) and COVID-19 Peak 2 (1 ± 2), with *p* < 0.001. There was no difference in patient demographics, symptoms at presentation, type of management, and timing of surgical management.

**Conclusion:**

The COVID-19 restrictions led to a major shift in the way healthcare was delivered, with increased use of telephone consultations and prompt early discharge from the hospital. Although we did not record safety issues or adverse outcomes, we found a delay in gestational age at presentation and diagnosis, which has the potential to lead to adverse outcomes. The COVID-19 pandemic has further highlighted the importance of telemedicine in healthcare practice.

## INTRODUCTION

Coronavirus disease 2019 (COVID-19) is a highly contagious pneumonia caused by the severe acute respiratory syndrome coronavirus 2.^1^ The emerging COVID-19 pandemic sent shock waves through the global health system, significantly disrupting care provision with high levels of morbidity and mortality worldwide.^2^

As the devastating impact of the disease became evident in the community, along with the rising number of COVID-19-related hospital admissions, specific preventative measures to limit the spread of the disease, such as physical distancing, face masks in public areas, and self-isolation, were introduced. Across the world, routine elective surgeries were initially postponed and later cancelled with increased emphasis on making operating rooms readily available to be converted into intensive care units to create capacity for critically ill COVID-19 patients.^2^ In Qatar, like many countries worldwide,^3,4^ a large COVID-19 treatment center was established to increase capacity, fully equipped with state-of-the-art intensive care equipment, staffed with intensivists, nurses and other healthcare professionals deployed from other facilities across the country. Additionally, another local hospital was converted into a COVID-19 treatment center for pregnant women who have been diagnosed with the virus. Furthermore, the traditional face-to-face clinic consultation was replaced by telemedicine where appropriate. These measures, combined with the fear of contracting COVID-19 in hospitals, resulted in a substantial decline in the number of women presenting themselves to emergency department (ED) services.^5–7^

In addition to patient reluctance to attend the hospital, institutions have implemented measures to minimize unnecessary patient contact.^2,6,7^ There were perceived risks of increased transmission during surgery, with studies reporting significantly higher rates of non-surgical management during the first wave of COVID-19 compared to the pre-pandemic cohort, along with fewer hospital visits among those managed non-surgically.^8^

This study aimed to compare the impact of the COVID-19 pandemic on gynecological emergency care with particular reference to early pregnancy complications (ectopic and miscarriage) during pre-pandemic periods with the first and second COVID-19 waves in Qatar.

## METHODS

### Setting and design

This was a retrospective, single-center, cross-sectional study conducted at the Women’s Wellness and Research Center (WWRC), a large teaching hospital in Qatar with more than 18,000 deliveries annually. Approval for the study was granted by the Hamad Medical Corporation Institutional Review Board (MRC-01-22-357) before conducting the study, with a waiver of consent for data extraction and analysis. The research was conducted in full compliance with the principles of Good Clinical Practice and accordance with the laws and regulations of the Ministry of Public Health in Qatar. The STROBE checklist was used to prepare this manuscript.^9^

## PARTICIPANTS

The study encompassed the first 50 women attending the ED of WWRC with a gynecological complaint of early pregnancy complications during the first COVID-19 peak in June 2020 (Peak 1) and the first 50 women during the second COVID-19 peak in April 2021 (Peak 2). Fifty women during the pre-COVID-19 period in 2019, who presented to the ED in the same time period of the year, were identified as the control group (pre-COVID-19). As the aim was to have a quick understanding of the impact of COVID-19 on emergency gynecology services, these numbers were considered adequate to evaluate the impact of COVID-19 restrictions on hospital services.

### Data source

After identifying all cases that met the inclusion criteria during the three time periods, data extraction was performed using the hospital’s electronic patient record system. The demographic details of each participant, including age, parity (number of previous deliveries after 24 weeks) categorized as nulliparous, multiparous 1–4 deliveries and grand multiparous (> 4 deliveries), gestational age at diagnosis, and nationality (divided into three groups: Qatari, Arab, and Non-Arab), were collected.

A descriptive comparison was made of the three time periods in terms of key performance indicators of the ED to ascertain the impact of COVID-19 restrictions on early pregnancy emergency services at the hospital. Clinical presentation, management (medical or surgical), and any patient safety issues in terms of complications of early pregnancy (miscarriage and ectopic pregnancy) were compared during the three periods to determine the impact of the COVID-19 restrictions on patient care.

### Statistical analysis

Data from the three groups (pre-COVID-19, COVID-19 Peak 1, and COVID-19 Peak 2, as previously defined) were analyzed and compared for patient demographics and the impact of COVID-19 on diagnosis and treatment of ectopic pregnancy and miscarriage. Continuous variables were represented as the mean and standard deviation (SD) or the median and interquartile range (IQR), depending on the distribution of the variable. Comparisons were performed using the ANOVA test or the Kruskal-Wallis test, as appropriate. Categorical variables were represented as frequencies and percentages and compared using the Chi-square test. Comparative or inferential statistics was done using Stata statistical software (version 18).^10^ The significance level was set at *p* < 0.05.

## RESULTS

Fifty participants were included in each study period, for a total of 150 participants. The demographics are shown in Table 1. There was no statistically significant difference in the mean age and parity of the three groups. The gestational age at diagnosis (weeks) was significantly greater in COVID-19 Peak 1 (Mean &x0026; SD; 12.4 ± 4.0) versus 10.9 ± 3.6 in the pre-COVID-19 period and 9.7 ± 3.9 in COVID-19 Peak 2 (*p* < 0.002). There was no statistically significant difference in the nationalities (Qatari, Arab, and non-Arab) and parity of the women in the three groups. There were more ectopic pregnancies presented to the ED in COVID-19 Peak 2 period compared to pre-COVID-19 and COVID-19 Peak 1 periods (*p* = 0.006). Most of the women in the three groups presented to the ED during the first trimester, with miscarriage being the most common diagnosis (*p* = 0.07). Only 2 of the 150 women were diagnosed with molar pregnancy.

There was no statistically significant difference in the number of ruptured and unruptured ectopic pregnancies between the three groups, as shown in Table 2. Pain was the main presenting symptom in all three groups, and there was no statistically significant difference in the number of women who underwent laparoscopic surgery between the three groups. During COVID-19 Peak 2, the number of women receiving medical management was twice that of the combined figures from the pre-COVID-19 period and COVID-19 Peak 1. Four women in the COVID-19 Peak 2 had blood transfusions compared to one in COVID-19 Peak 1, and none in the pre-COVID-19 period.

The mean (SD) gestation age (weeks) for miscarriages in COVID-19 Peak 1 was 13.2 ± 3.7 compared to 11.4 ± 3.4 in the pre-COVID-19 period and 11.4 ± 3.7 in COVID-19 Peak 2 as shown in Table 3. Although there was no statistically significant difference in symptoms at presentation between the study periods, more women presented with bleeding during the three periods compared to pain or being asymptomatic. There was no statistically significant difference in surgical or medical management between the three groups. The length of hospital stay was statistically longer in the pre-COVID-19 period (2 ± 1.5) versus 1 ± 2 in the COVID-19 Peak 1 and COVID-19 Peak 2 periods (*p* < 0.001).

## DISCUSSION

As the effects of the pandemic impacted every aspect of society and governments worldwide implemented restrictive measures, healthcare professionals worldwide reported a significant decrease in the number of patients visiting the ED, as well as an increased use of non-surgical options for gynecological emergencies.^5,6^ Spurlin et al.^11^ reported that, although there was a 60% decrease in the volume of overall obstetrics and gynecology ED consultations, there was no change in the percentage of patients in the ED who required ED consultations. Furthermore, they found no significant changes in hospital admissions, blood transfusions, and emergency surgery. Conversely, Anteby et al.^8^ reported that more women presented with ruptured ectopics and received blood transfusions during the COVID-19 period compared to the non-COVID-19 period, with a similar number of cases treated surgically. Platts et al.^12^ reported that a higher number of women in their study underwent non-surgical management. In our study, we have focused on the two most common early pregnancy complications (ectopic pregnancy and miscarriage) that require either medical or surgical management. We did not compare the ED attendance rate during the three study periods due to the implementation of numerous restrictive measures that limited visits to patients deemed by physicians to require face-to-face consultations or considered “true emergencies”. Despite this, we found no difference in those undergoing surgical or medical management for early pregnancy emergencies during the study periods.

In this study, we found no difference in patient demography, clinical presentation, and type of management for early gynecological problems for both ectopic pregnancy and miscarriage between the three groups. This observation is consistent with the findings of a systematic review on the management and complications of ectopic pregnancy during the pandemic by Morin et al.,^5^ who reported no difference in the type of management for ectopic pregnancy between the pre-COVID-19 and COVID-19 periods.

We observed a significant delay in gestational age at diagnosis during the COVID-19 Peak 1 compared to pre-COVID-19 and COVID-19 Peak 2 periods. It is difficult to determine whether this delay in diagnosis was due to the patient’s adherence to self-isolation directives, reluctance to come to the hospital for fear of contracting COVID-19, or delay in patients getting early dating ultrasound appointments due to restrictive measures that limit the number of patients in hospital premises at any given time. It is also possible that, as a better understanding of the disease became apparent within the community following the COVID-19 Peak 2, people became less fearful of contracting the disease, resulting in patients being more willing to seek prompt medical attention.

Although we did not observe any statistically significant difference in the management of miscarriage and ectopic pregnancy during the study periods, we noticed a significantly shorter length of hospital stay during COVID-19 Peak 1 compared to pre-COVID-19 and COVID-19 Peak 2 periods. Possible explanations for the decreased length of hospital stay include doctors’ desire to discharge patients as soon as they are fit for discharge and patients’ reluctance to stay in the hospital environment due to fear of contracting COVID-19.

We noticed a significant increase in the number of ectopic cases diagnosed during the COVID-19 Peak 2 period. This may be directly reflective of the restriction policies that would have reduced the number of women with asymptomatic first-trimester miscarriages approaching the ED, leading to the inclusion of a higher number of ectopic pregnancies in our sample for COVID-19 Peak 2. Despite the restrictive measures and increased use of telemedicine during the COVID-19 period, we found no increase in complications or adverse outcomes during these periods. Similarly, Platts et al.^12^ reported no increase in complications among ectopic pregnancy cases treated during the first wave of COVID-19 compared to the pre-pandemic period, despite the significantly higher number of women undergoing non-surgical management and having fewer hospital visits. These findings may represent indirect evidence that some of our prehistoric practices are inappropriate and require review to ensure the more cost-effective use of emergency resources.

### Strengths and limitations

A major strength of the study is that it covered a 3-year period, encompassing the pre-COVID-19 year, COVID-19 Peak 1, and COVID-19 Peak 2. To our knowledge, this is the first study to compare the three periods with a specific focus on early pregnancy complications, providing valuable insights into the impact of health restrictions on the management of first-trimester pregnancy complications. This study has certain limitations. First, this is a retrospective study, and as such, it is associated with intrinsic biases. This is a single-center study with a small sample size; the small subgroup analysis makes it challenging to detect statistically significant differences, and therefore, the findings cannot be generalized to the broader population. The small clinical implications and patient satisfaction with telemedicine need further evaluation through larger and long-term studies.

## CONCLUSION

The COVID-19 restrictions led to a significant shift in the way healthcare was delivered, with an increased use of telemedicine and a trend toward prompt early discharge from the hospital. Although we did not record any safety issues or adverse outcomes, we found a delay in gestational age at presentation and diagnosis, which has the potential to lead to adverse outcomes. COVID-19 has further highlighted the importance of telemedicine in healthcare practice. Notably, healthcare professionals remain vigilant at all times to ensure that patient welfare and safety are not compromised.

## List of abbreviations

**Table:** 

ANOVA	Analysis of variance
COVID-19	Coronavirus disease 2019
ED	Emergency department
HMC	Hamad Medical Corporation
IQR	Interquartile range
SARS-CoV-2	Severe acute respiratory syndrome coronavirus 2
SD	Standard deviation
WWRC	Women’s Wellness and Research Center

## Acknowledgments

We would like to thank our front-line healthcare workers in the emergency department, including physicians and nurses, who handled the workflow during the COVID-19 pandemic with extreme dedication and often at personal risk. This study would not have been possible without their efforts and input.

## Conflicts of interest

The authors declare no conflicts of interest.

## Figures and Tables

**Table 1.** Patient demographics between the 3 years.

**Patient demographics**		**2019 (Pre-COVID-19) *N* = 50**		**2020 (COVID-19 Peak 1) *N* = 50**		**2021 (COVID-19 Peak 2) *N* = 50**		***p* value**
Age in years (Mean ± SD)		31.3 ± 5.5		32.7 ± 5.5		33.3 ± 4.9		0.162
Parity (Median ± IQR)		2 ± 3		2 ± 3		1.5 ± 2		0.380^#^
Gestational age (weeks) at presentation (Mean ± SD)		10.9 ± 3.6		**12.4 ± 4.0**		9.7 ± 3.9		0.002*

		***n***	**%N**	***n***	**%N**	***n***	**%N**	***p* value**

Nationality	Qatari	13	26.0	15	30.0	15	30.0	0.636
	Arab	22	44.0	20	40.0	15	30.0	
	Non-Arab	15	30.0	15	30.0	20	40.0	
Parity	Nulliparous	14	28.0	12	24.0	12	24.0	0.278
	Multiparous	28	56.0	21	42.0	28	56.0	
	Grand Multiparous	8	16.0	17	34.0	10	20.0	
Gestational age at presentation	First trimester (<12 weeks)	41	82.0	34	68.0	43	86.0	0.07
	Second trimester (12–23 weeks + 6 days)	9	18.0	16	32.0	7	14.0	
Diagnosis	Ectopic	6	12.0	6	12.0	**17**	**34.0**	0.006*
	Miscarriage	44	88.0	42	84.0	33	66.0	
	Molar	0	0	2	100.0	0	0	

^#^Comparison using Kruskal Wallis test, Other continuous variables compared using ANOVA, SD: standard deviation, IQR: interquartile range; Categorical variables compared using Fisher’s exact test.

*p value < 0.05. The values in bold represent the observations that are significantly different.

**Table 2.** Comparison of ectopic pregnancy between the groups.

**Variables for ectopic**		**2019 (Pre-COVID-19) *N* = 6**		**2020 (COVID-19 year 1) *N* = 6**		**2021 (COVID-19 year 2) *N* = 17**		***p* value**
		***n***	**%*N***	***n***	**%*N***	***n***	**%*N***	
Type of ectopic	Ruptured	3	50.0	2	33.3	7	41.2	1.00
	Unruptured	3	50.0	4	66.7	10	58.8		
Gestational age (weeks) at presentation (Mean ± SD)		7.2 ± 3.1		7.7 ± 2.2		6.4 ± 1.1		0.301
Symptoms at presentation	Asymptomatic	2	33.3	1	16.7	2	11.8	0.553
	Pain	4	66.7	5	83.3	14	82.4	0.820
	Bleeding	1	16.7	4	66.7	7	41.2	0.199
Time to surgical intervention	No theatre	2	33.3	2	33.3	9	52.9	0.704
	<24 hours	3	50.0	4	66.7	6	35.3	
	>24 hours	1	16.7	0	0	2	11.7	
Management	Laparotomy	0	0	1	16.7	1	16.7	0.867
	Laparoscopy	4	66.7	3	50.0	7	41.2	
	Methotrexate	2	33.3	2	33.3	8	47.1	
Blood transfusion		0	0	1	16.7	4	23.5	0.794
Number of days admitted (Median ± IQR)		1 ± 1		2 ± 0		1 ± 1		0.220^#^
Estimated blood loss (ml) (Median ± IQR)		125 ± 280		40 ± 50		20 ± 180		0.755^#^
Haemoglobin at discharge (Mean ± SD)		10.9 ± 1.6		12.5 ± 1.0		11.2 ± 1.5		0.138

^#^Comparison using Kruskal Wallis test, Other continuous variables compared using ANOVA, SD: standard deviation, IQR: Interquartile range; Categorical variables compared using Fisher’s exact test.

**Table 3.** Comparison of miscarriages between the years.

**Variables for miscarriage**		**2019 (Pre-COVID-19) *N* = 44**		**2020 (COVID-19 year 1) *N* = 42**		**2021 (COVID-19 Peak 2) *N* = 33**		***p* value**
		***n***	**%*N***	***n***	**%*N***	***n***	**%*N***	
Type of miscarriage	Incomplete	21	47.7	19	45.2	11	33.3	0.257
	Missed	13	29.6	17	40.5	17	51.5	
	Others	10	22.7	6	14.2	5	15.2	
Gestational age (weeks) at presentation (Mean ± SD)		11.4 ± 3.4		13.2 ± 3.7		11.4 ± 3.7		0.036*
Gestational age at presentation	First trimester (<12 weeks)	35	79.6	26	61.9	26	78.8	0.142
	Second trimester (12–23 weeks + 6 days)	9	20.4	16	38.1	7	21.2	
Symptoms at presentation	Asymptomatic	16	36.4	7	16.7	7	21.1	0.105
	Pain	14	31.8	28	66.7	17	51.5	0.005*
	Bleeding	26	59.1	32	76.2	24	72.7	0.197
Time to surgical intervention	No theatre	26	59.1	23	54.8	16	48.5	0.877
	<24 hours	7	15.9	9	21.4	8	24.2	
	>24 hours	11	25.0	10	23.8	9	27.3	
Management	Nil	3	6.8	7	16.7	2	6.1	0.591
	Medical	23	52.3	15	35.	14	42.2	
	Medical + Surgical	11	25.0	11	26.2	11	33.3	
	Surgical	7	15.9	8	21.4	6	18.2	
Blood transfusion		3	6.8	5	11.9	2	6.1	0.648
Number of days admitted (Median ± IQR)		2 ± 1.5		1 ± 2		1 ± 2		<0.001*^#^
Estimated blood loss (ml) (Median ± IQR)		50 ± 75		100 ± 280		50 ± 130		0.116^#^
Haemoglobin at discharge (Mean ± SD)		11.0 ± 1.6		10.8 ± 1.9		11.1 ± 1.6		0.778

^#^Comparison using Kruskal Wallis test, Other continuous variables compared using ANOVA, SD: standard deviation, IQR: Interquartile range; Categorical variables compared using Fisher’s exact test.

*p value < 0.05. The values in bold represent the significant p values at p < 0.005.
